# Analysis of genetic variation in Ashkenazi Jews by high density SNP genotyping

**DOI:** 10.1186/1471-2156-9-14

**Published:** 2008-02-05

**Authors:** Adam B Olshen, Bert Gold, Kirk E Lohmueller, Jeffery P Struewing, Jaya Satagopan, Stefan A Stefanov, Eleazar Eskin, Tomas Kirchhoff, James A Lautenberger, Robert J Klein, Eitan Friedman, Larry Norton, Nathan A Ellis, Agnes Viale, Catherine S Lee, Patrick I Borgen, Andrew G Clark, Kenneth Offit, Jeff Boyd

**Affiliations:** 1 Departments of Epidemiology and Biostatistics, Memorial Sloan-Kettering Cancer Center, New York, NY, USA; 2 Surgery, Memorial Sloan-Kettering Cancer Center, New York, NY, USA; 3 Medicine, Memorial Sloan-Kettering Cancer Center, New York, NY, USA; 4 Programs in Cancer Biology and Genetics, Memorial Sloan-Kettering Cancer Center, New York, NY, USA; 5 Molecular Biology, Memorial Sloan-Kettering Cancer Center, New York, NY, USA; 6 Laboratories of Genomic Diversity, National Cancer Institute, Bethesda, MD, USA; 7 Population Genetics, National Cancer Institute, Bethesda, MD, USA; 8 Department of Molecular Biology and Genetics, Cornell University, Ithaca, NY, USA; 9 Department of Computer Science and Engineering, University of California, San Diego, La Jolla, CA, USA; 10 Chaim Sheba Medical Center, Tel-Hashomer, and Sackler School of Medicine, Tel-Aviv University, Tel-Aviv, Israel; 11 Department of Medicine, University of Chicago, Chicago, IL, USA

## Abstract

**Background:**

Genetic isolates such as the Ashkenazi Jews (AJ) potentially offer advantages in mapping novel loci in whole genome disease association studies. To analyze patterns of genetic variation in AJ, genotypes of 101 healthy individuals were determined using the Affymetrix EAv3 500 K SNP array and compared to 60 CEPH-derived HapMap (CEU) individuals. 435,632 SNPs overlapped and met annotation criteria in the two groups.

**Results:**

A small but significant global difference in allele frequencies between AJ and CEU was demonstrated by a mean *F*_*ST *_of 0.009 (*P *< 0.001); large regions that differed were found on chromosomes 2 and 6. Haplotype blocks inferred from pairwise linkage disequilibrium (LD) statistics (Haploview) as well as by expectation-maximization haplotype phase inference (HAP) showed a greater number of haplotype blocks in AJ compared to CEU by Haploview (50,397 vs. 44,169) or by HAP (59,269 vs. 54,457). Average haplotype blocks were smaller in AJ compared to CEU (e.g., 36.8 kb vs. 40.5 kb HAP). Analysis of global patterns of local LD decay for closely-spaced SNPs in CEU demonstrated more LD, while for SNPs further apart, LD was slightly greater in the AJ. A likelihood ratio approach showed that runs of homozygous SNPs were approximately 20% longer in AJ. A principal components analysis was sufficient to completely resolve the CEU from the AJ.

**Conclusion:**

LD in the AJ versus was lower than expected by some measures and higher by others. Any putative advantage in whole genome association mapping using the AJ population will be highly dependent on regional LD structure.

## Background

As a result of the completion of the HapMap [1], and the development of platforms for high-throughput SNP genotyping, it is now possible to carry out genome wide association studies to map novel disease associated loci [2-6]. Typing a dense panel of SNPs across the genome permits the identification of genetic variants associated with disease. It has been proposed that the study of "founder populations" derived from genetic isolates may improve the efficiency of mapping polygenic traits [7]. One example of a genetic isolate is Ashkenazi Jews (AJ), where the combination of random genetic drift and at least three population "bottlenecks" resulted in a limited number of founder mutations associated with common inherited diseases [8-10]. At the outset of these studies, our working hypothesis was that the younger overall age of such populations should permit a smaller number of SNPs to explain the overall genetic variability.

In the AJ population, in disease bearing individuals, haplotype blocks, or regions of linkage disequilibrium (LD) associated with disease, are indeed large, on the order of one to10 Mb in the regions immediately surrounding founder mutations. AJ disease with these characteristics includes hereditary breast and ovarian cancer [11,12]. Therefore, we reasoned that a systematic genomic analysis of LD structure in AJ would facilitate genome wide association studies to identify cancer-susceptibility loci in AJ. Consequently we set to better understand patterns of genetic variation in this group, including allele frequency spectra, deviations from Hardy-Weinberg proportions, differences in allele frequencies from other European populations, including patterns of LD.

## Methods

### Study population

The study enrolled 102 healthy AJ women who either accompanied male urology patients identified through the Urology Clinic or who were participating in cancer screening at the Memorial Sloan-Kettering Cancer Center (MSKCC). Participants completed a self-administered questionnaire about their medical history, date of birth, date of last mammogram, race, religious affiliation, as well as country of birth and religious affiliation of grandparents. To be eligible for enrollment in this study, individuals must have indicated that all four grandparents were Jewish and of Eastern European ancestry. Any woman who indicated a prior diagnosis of breast cancer, atypical hyperplasia, or lobular carcinoma in situ was not eligible for this study. Informed consent and blood specimens were obtained from these women under an IRB-approved protocol at MSKCC. One research subject withdrew permission to use her DNA prior to laboratory analysis. That sample and related records were redacted from the study. The remaining subjects enrolled in this study were the first 101 individuals of a planned ascertainment of 300 control individuals from another ongoing study.

### AJ genotyping

Preparation of genomic DNA from blood was performed as previously described [13]. Genotyping was carried out using Affymetrix GeneChip Early Access Version 3 (EAv3) Human Mapping Arrays. Use of Affymetrix EaV3 chips for genotyping was performed as described in the Gtype 4.0 manual [14] except that 150 ng of all genomic DNA samples were evaluated for quality by gel electrophoresis. Following qualification of the DNA samples, each sample was divided into two aliquots. Sequence complexity was reduced by restriction enzyme digestion with either *Nsp*I or *Sty*I, and a biotin-labeling primer amplification assay was performed on each DNA aliquot. Hybridization of the amplified probes was then performed on specific *Nsp*I or *Sty*I arrays, as appropriate.

Microarray output was analyzed using the Bayesian robust linear modeling using Mahalanobis distance (BRLMM) algorithm [15]. This is a modification of a published algorithm that normalizes fluorescent signals across multiple chips and makes inference across multiple SNPs to render more accurate calls [16]. In addition, the Bayesian component helps to reduce the bias against heterozygote calls (relative to the prior dynamic modeling algorithm). Only those 435,632 SNPs present both on the EAv3 and the Affymetrix 500 K commercial array that had dbSNP rs numbers were included in further analyses. Of these, 221,233 SNPs were present on the *Nsp*I array and 214,399 SNPs were present on the *Sty*I array. Mapping of the SNPs to the May 2004 (NCBI build 35, hg 17) coordinates was possible through the annotation files supplied by Affymetrix [17].

The distribution of SNPs on each chromosome arm (Additional File 1, Table S1) was approximately proportional to the size of the arm, with the exceptions of arms 19p, Xp, and Xq, each of which had fewer than 80 SNPs per Mb compared to the average of 163 SNPs per Mb for the other chromosome arms. The other exception was chromosome 21p, which was represented by a total of only 4 SNPs. For the 43,998,832 attempted genotype calls, a total of 511,653 were no calls (1.1%). Overall, the percentage of no calls per SNP ranged from 0% to 72% (SD, 2.7%) (Additional File 2, Fig. S1). The percentage of no calls per SNP ranged from 0% to 72% (SD, 2.9%) for the *Nsp*I array, and 0% to 50%, (SD, 2.4%) for the *Sty*I array. With respect to the two arrays (Additional File 3, Fig. S2), the percentage of no calls for the *Nsp*I array ranged from 0.4% to 4.6% (SD, 0.6%) and the percentage of no calls for StyI array ranged from 0.2% to 4.5% (SD, 0.7%). Every statistic presented here was recalculated for a more limited set of SNPs (167,676) that had completion rates of 99.6% or above.

### CEU genotyping

Data from 60 CEPH-derived CEU subjects that were also part of the HapMap Project [18] were included in the analysis for reference. These 60 individuals were the parents of 30 trios, with the children excluded so that the genotype data could be considered independently. These experiments were performed by Affymetrix [19]. As with the AJ population, CEU genotypes were determined using the BRLMM algorithm. Of 26,137,920 attempted genotypes on the CEU population, there were 145,402 (0.6%) no calls, lower than the1.1% no call rate for the AJ (Additional File 4, Fig. S3). The percentage of no calls by SNP ranged from 0% to 68% (SD, 1.7%), with a range of 0% to 43% (SD, 1.8%) for the *Nsp*I array and 0% to 68% (SD, 1.5%) for the *Sty*I array. With respect to the two arrays (Additional File 5, Fig. S4), the percentage of no calls ranged from 0.1% to 2.7% (SD, 0.7%) for the *Nsp*I array and 0.1% to 2.4% (SD, 0.4%) for the *Sty*I array. For each of the 90 CEU individuals, there were 489,913 genotype calls available from the HapMap web site (HapMap version 20, July, 2006). When compared with the commercial Affymetrix chip, there was 99.4% concordance (43,638,269 genotype calls agreed). In making this assessment, there were 67,394 absolutely discordant genotype calls (called heterozygous in HapMap but homozygous by Affymetrix array or vice-versa), leading to mean call confidence scores for AJ and CEU that were within 3% of each other but significantly different. These differences and slight discordance probably reflects the large number of SNPs analyzed and use of the Affymetrix commercial arrays for the CEU analysis and early release arrays for the AJ analysis.

### Determination of SNP frequencies and Hardy-Weinberg departures

Several SNP characteristics were compared between the AJ and CEU subjects. Minor allele frequencies for each SNP were compared between populations using Fisher's exact test, with the Bonferroni method used to adjust for multiple comparisons. Global patterns in allele frequency were examined using the fixation index of the subpopulation within the total (*F*_*ST*_) [20] and the Wilcoxon signed rank test to assess significance. The Hardy-Weinberg disequilibrium coefficient (*D*_*A*_) was calculated in order to assess both magnitude and direction of departures from Hardy-Weinberg equilibrium (HWE) [20]. It is noted that the sign of *D*_*A *_for any departure is precisely the same as the sign of the fixation index for the individual within the subpopulation (*F*_*IS*_), which can be derived from *D*_*A *_by a simple algebraic manipulation. Exact tests of HWE were computed as described [21]. Regions out of HWE were identified using circular binary segmentation (CBS) with a chi-square statistic instead of a *t*-statistic [22]. This recursive algorithm was applied one chromosome at a time. The rates of heterozygosity were also computed, as was *F*_*ST*_. Also computed were Nei's [23] standard genetic distance measure (*D*_*s*_) and six recent implementations of the information theoretic measures. These included entropy for admixed populations, assuming three different admixture percentages: 10%, 50% (maximum admixture) and 90%. Additionally, three information theoretic measures that may be useful for differentiating individuals within populations on the basis of selected SNPs were computed: two forms of Kullback-Leibler divergence and an informativeness for assignment statistic suggested by Rosenberg et al. [24]. The results from these last two sets of analyses are available on our public browser described in a subsequent section. For the X-chromosome analyses of HWE and computation of genetic measures, only the 30 females in the CEU data set were included.

### Linkage disequilibrium and haplotypes

A major argument for using founder populations for genetic mapping is that they may be expected to display greater levels of LD and lower haplotype complexity. In order to test the former hypothesis, a subset of SNPs passing HWE criteria and having a minor allele frequency of greater than 10% in both populations was identified. The standard LD estimators *r*^2 ^and D' were calculated, restricting the analysis to SNP pairs within 500 kb of one another. Since both estimators are influenced by sample size, the same number of AJ and CEU individuals were compared. To accomplish this, six sets of 60 AJ individuals were randomly selected from the 101 total AJ individuals. A nonparametric sign test was used to test significance of the difference in LD between the CEU and AJ. For each subset of 60 AJ and 60 CEU, the test was performed on the total set of SNP pairs, on a subset of SNP pairs where each SNP was tested only with its nearest neighbor (if a SNP did not have a neighbor within 40 kb, that SNP was dropped), and to examine longer range LD, on a subset of SNP pairs where each SNP was tested with another SNP approximately 200 kb away. This same sign test was also used in 1 Mb windows in order to identify local regions of the genome showing exceptional differences in LD patterns. In addition, the decay of LD over distance was examined by plotting average *r*^2 ^between SNP pairs across a wide range of distances from 0–5, 5–10, out to 475–500 kb apart. Here, *r*^2 ^was averaged over the six sub-samples of 60 AJ and 60 CEU.

As an additional metric to compare LD between the two populations, the proportion of SNP pairs with no evidence of recombination (*D' *= 1.0) at different distance intervals between the SNPs was examined (the same described above for the decay of *r*^2^). The same sample sizes of 60 AJ and 60 CEU were used, and averaged across the six replicates.

Haplotype blocks were estimated in two ways. The first was by using the HAP program to infer linkage phase relationships and reconstruct chromosomal haplotypes [25,26]. This algorithm determines local haplotypes by exploiting the correlation between SNPs in physical proximity resulting from LD using a genealogy based model of perfect phylogeny. The reconstructed chromosomal haplotypes were partitioned into blocks of limited diversity. A haplotype block is defined here as a span of SNPs such that haplotypes with a minimum frequency of 5% account for at least 80% of the haplotypes in a block. Separate block partitions for samples in each of the two populations were determined. (HAP derived haplotype blocks by chromosome are available upon request from the authors.) Another haplotype block definition utilized was the default settings of Haploview [27], which uses pair-wise LD statistics to implement the methods of Gabriel et al. [28]. One key difference between HAP and Haploview is that the former assigns every SNP to a haplotype, while the latter constructs haplotype blocks based only on pairwise LD. Triangle diagrams derived from the Haploview data are available [29].

### Homozygosity mapping

Homozygous tracts were defined using an extension of the likelihood-based method originally applied to short tandem repeat polymorphisms [30]. A log likelihood odds (lod) score was calculated at each SNP, contrasting the likelihood of observing the genotype assuming the surrounding segment is autozygous (as opposed to not autozygous). The method accounts for population-specific allele frequencies as well as for genotyping error and mutation (combined term, ε = .005). Since SNPs are not independent, the lod score was adjusted using a log-transformation of a recombination measure (rec_val, in cM/kb, from the Oxford HapMapI r16c.1 UCSC tract) at each SNP position, according to the formula (1 + log_10_(1 – (0.39 – rec_val))). For every run of two or more consecutive homozygous SNPs in an individual, the adjusted lod scores were summed and a homozygous tract was defined when the score reached 20. Homozygous tracts were not disrupted by SNPs with missing information ("no call"), which did not contribute to the score, and tracts were not allowed to cross centromeres or gaps in the human genome assembly of estimated size of 50 kb or greater. To compare populations, the proportion of the genome within homozygous tracts and the tract length (kb) were compared using the Wilcoxon rank sum test (two-sided Z approximation).

### Additional data sets examined

Subsequent to detailed examination of the 101 AJ and 60 CEU genotype data sets just described, we sought to verify our results: For this purpose, we culled any SNP with more than two no calls based on a theoretical optimization of our heterozygosity detection. This resulted in a dataset of 167,676 SNPs on which we performed each analysis. In addition, we carried-out a cross-examination with additional data. For this purpose, we examined the ~317,000 genotypes from 151 Jewish women included in a recent study [31]. We ran HaploView on this dataset using the default parameters previously described. The results of each of these validation data sets are displayed on our public browser described in a subsequent section.

### Principal components analysis

In order to determine whether the two populations were sufficiently differentiated to resolve them based on examination of these SNP sets, we applied the numerical methods implemented in the Eigenstrat package [32]. Rather than use the second half of the package to adjust association p-values for population stratification, we used only the Principal Components analysis portion (pca) of the package and graphed the result of Component 1 versus Component 2. In addition to the graph presented, we applied this pca to both the data described in a recent study [31], and to the data in the SNP set reduced to minimize no calls (the 167,676 SNPs described above). Each set of data provided comparable results. To confirm all derived indices measuring excess heterozygosity, rate of LD decay over genetic distance and measures of global patterns of allele frequency difference such as *F*_*ST*_, we recalculated these measures on the cohort of 87 AJ subjects that remained after 14 outliers detected by pca were removed.

### Browser and software

The generic genome browser, gbrowse [33], was used to create a visual display of project data. This visual display is publicly available and can be accessed from the main page of that URL under the rubric "Projects" [34]. The data on that browser include: 1) call consistency measures based on mean Wilcoxon's signed rank test *P*-values for BRLMM call confidence; 2) Hardy-Weinberg compliance statistics; and 3) human population genetic distance measures. The R-statistical package was used extensively for summary graphics. Data analyses were performed with SAS, SAS/Genetics, SPSS or R, unless otherwise noted.

## Results

### SNP characteristics

**Figure 1:**
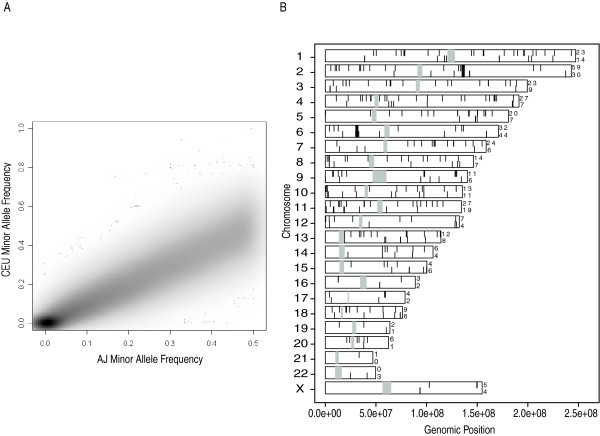
Comparison of minor allele frequencies between AJ and CEU. **a**, Allele frequencies were computed for AJ first, and major and minor allele were assigned on the basis of which allele predominated. Therefore, no minor allele in AJ had a frequency greater than 0.50. Minor allele frequencies were plotted using the RcolorBrewer portion of the geneplotter library of Bioconductor in R. Default values were used for the smoothing. All 435,632 SNPs common to AJ and CEU were plotted. **b**, Karyogram highlighting markers with significantly different allele frequencies between AJ and CEU. Positions of significance are identified with black lines and centromeres are identified by gray. Lines drawn from the top indicate a higher minor allele frequency in AJ lines drawn from the bottom indicate a higher minor allele frequency in CEU. Counts on the right are the number of significant markers on the chromosome for AJ and CEU. The cutoff for significance is a Bonferroni-adjusted *P*-value of less than 0.05. Dense lines on chromosomes 2 and 6 are evidence for regions of gross allele frequency differences.

There were 39,664 (9.1%) monomorphic loci in the AJ population and 54,170 (12.4%) in the CEU population, with a strong overlap; 35,269 (89.8%) of monomorphic AJ were monomorphic in both populations. Filtering these monomorphic loci from the allele frequency comparison did not appreciably change the shape or summary of allele frequency statistics. Scattergrams of the minor allele frequencies for both groups are shown in Fig. 1a. Although most points lie along the 22.5° line in the graph, there are some outliers. (They are expected to be on the 22.5° line instead of the 45° line because the major and minor alleles were determined based on the observed data for the AJ.) The mean minor allele frequency for the AJ was 0.21 (median 0.19). Pearson's correlation coefficient (R) for the minor allele frequency pairs between the two populations was 0.91. Spearman's rank correlation coefficient, which is appropriate for non-Gaussian data, yielded a similar value of 0.92.

**Figure 2:**
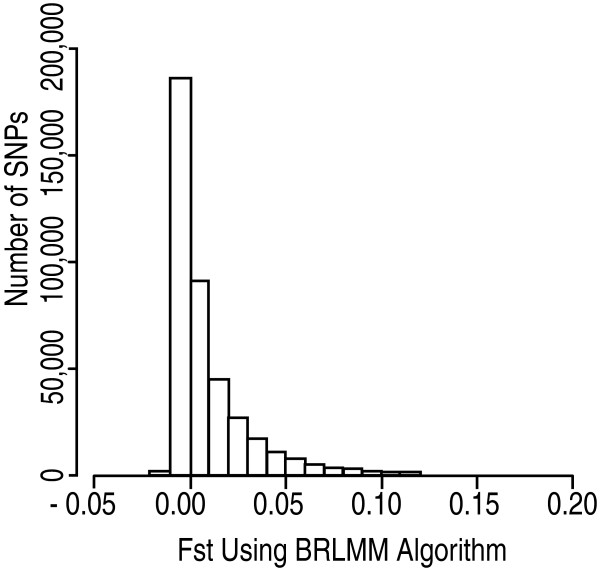
Histogram of *F*_*ST *_versus SNP number. Calculation of *F*_*ST *_for AJ and CEU was performed as described in the Materials and Methods section [20]. Outlier values with *F*_*ST *_> 0.2 were excluded from the histogram for purposes of clarity of visualization.

A comparison of allele frequencies revealed large regions that differ between the two groups on chromosomes 2 and 6 (Fig. 1b), consisting of a total of 533 SNPs. These chromosomes had local *F*_*ST *_values as high as 0.51 (chromosome 2) and 0.34 (chromosome 6). Mean *F*_*ST *_across the genome was 0.009 with a median of 0.001 (Fig. 2). While we consider this to be a small difference between the populations, it was statistically significant (*P *< 0.001). A permutation method was used to determine whether the observed *F*_*ST *_distribution differed significantly from the null. By permuting the ethnic label but retaining the SNP data, 100 datasets of pseudo-AJ and pseudo-CEU were created. Calculation of *F*_*ST *_statistics for the permuted set was then performed 100 times, along with calculation of mean, median, mode, variance, and 95% confidence intervals. Although additional permutations beyond 100 datasets could considerably diminish the *P*-value estimate, this method resulted in the lowest possible *P*-value (< 0.01), and thus rejection of the null hypothesis. By every metric examined, the AJ and CEU sample data were inconsistent with those expected by two draws from a single population.

The proportion of heterozygous SNPs was very similar between the two populations. For AJ, the mean per SNP was 0.28, the median was 0.31, and the SD was 0.18. For CEU, the mean per SNP was 0.28, the median was 0.30, and the SD was 0.18.

**Figure 3:**
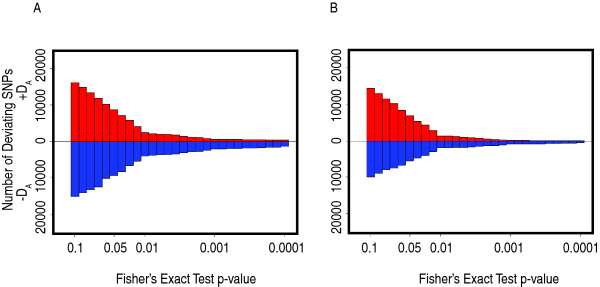
Horizontal back-to-back histograms displaying the direction of Hardy-Weinberg departures across a range of bins of Fisher's Exact test *P*-values. The number of SNPs with a positive *D*_*A *_value (excess homozygotes) is graphed above the zero line in each graph. The number of SNPs with a negative *D*_*A *_value is graphed below the zero line in each graph. **a**, Data from AJ SNPs. **b**, Data from CEU SNPs.

Compliance with HWE was evaluated separately for the AJ and CEU populations. At a level of α = 0.05, the number of deviations from HWE was greater for AJ, with 18,073 (4.1%) of the SNPs out of HWE compared to 12,317 (2.8%) for CEU. Neither population had more SNPs out of HWE than expected. Among the SNPs out of HWE, AJ had a majority with negative *D*_*A *_(51.6%), indicating excess heterozygosity, while CEU had a majority with positive *D*_*A *_(57.4%), indicating excess homozygosity. Both groups had excess heterozygosity among those with HWE departure at α = 0.01 (data not shown). The direction of HWE departures across a range of *P-*values is shown in Fig. 3. Additional analysis of HWE departures is provided in Additional File 6, Fig. S5.

Calculation of *F*_*IS *_was used to further quantify departures of genotypic frequencies from panmixia. Mean *F*_*IS *_was -0.0124 for AJ and -0.0131 for CEU. These comparable negative *F*_*IS *_values are consistent with evidence of similar levels of outbreeding (isolate breaking) in both populations and also suggest that the above-mentioned homozygosity excess in CEU at α = 0.05 was not a distinguishing characteristic, a point further supported by the similarity of shape of the HWE violations in the two populations (Fig. 3).

**Figure 4:**
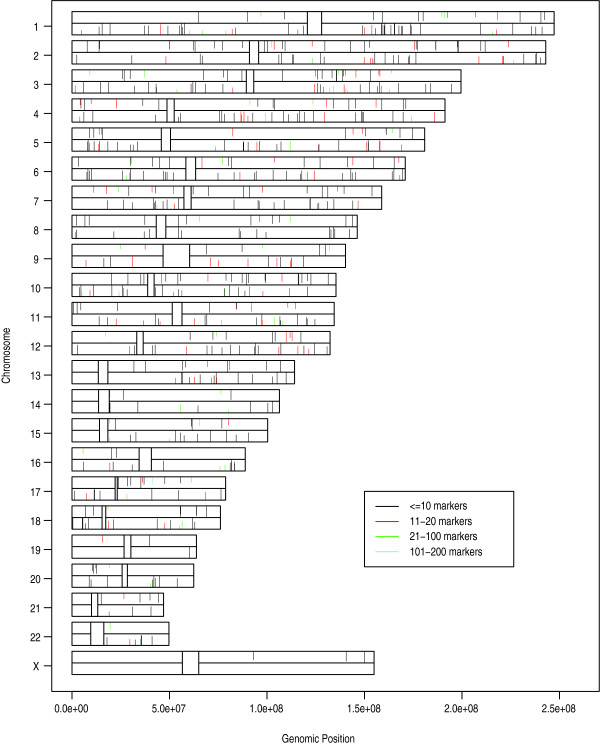
Karyogram of clusters of markers out of HWE. The lines from the top of each chromosome are from AJ and the lines from the bottom are from CEU. The closer a line is to the mid-point, the higher the proportion of markers in a cluster out of HWE at α = 0.05. Lines are drawn at the mid-point of the genomic positions of the clusters. Different colors correspond to different numbers of markers in a cluster. Centromeres are left blank. Clusters on the X-chromosome for CEU have been dropped because the CEU population consisted of half males and half females. Clusters were generated using the CBS algorithm [22].

An analysis was performed using the CBS algorithm in order to find clusters of markers out of HWE. The results indicate that, although the overall number of markers not in HWE was less than expected, there were small regions with all, or nearly all markers out of HWE (Fig. 4). Also notable is that there was very little overlap in the clusters between AJ and CEU.

### LD and haplotypes

**Figure 5:**
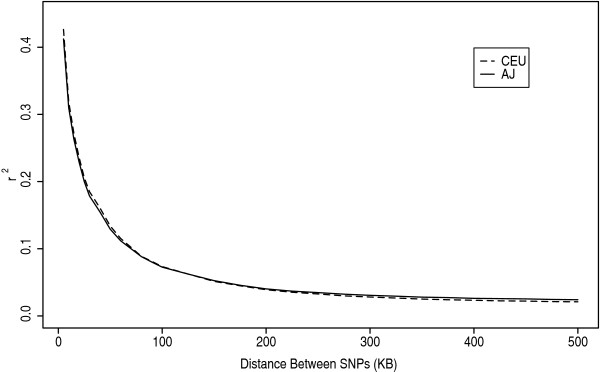
Decay of *r*^2 ^as a function of distance between pairs of SNPs on chromosome 1 for AJ and CEU. Plotted are the average *r*^2 ^values for pairs of SNPs within each bin. The values for AJ and CEU cross at ~200 kb.

The LD metric *r*^2 ^was calculated separately in AJ and CEU individuals for all SNP pairs within a 500 kb radius of one another. These tests were performed on subsets of 60 AJ individuals to match the sample size of the CEU dataset; had the full 101 AJ been compared to the 60 CEU, a spurious excess of LD would have resulted in the CEU [35]. Unless otherwise noted, the statistics that follow are averages across six such subsamples of AJ individuals. For bins of separation between the SNPs (0–5 kb, 5–10 kb, etc.), the mean *r*^2 ^was calculated for each bin (Fig. 5). These data revealed a pattern of LD decay as a function of distance, with AJ displaying a slightly more rapid rate of LD decay than CEU at short distances, but a slower rate of LD decay at distances beyond 200 kb. Patterns of LD decay measured by mean *D*' appeared qualitatively similar to those using *r*^2^.

Because metrics for LD could be calculated for the same pairs of SNPs in the two population samples, the simplest test comparing LD in AJ and CEU determined whether the count of cases where *r*^2^(AJ) > *r*^2 ^(CEU) vs. *r*^2^(AJ) <*r*^2^(CEU) corresponded to a binomial trial with *P *= 0.5. In the first test, the *r*^2 ^values compared were all those within a radius of 500 kb on chromosome 1. In this case, there were 1,067,034 SNP pairs where *r*^2 ^(AJ) > *r*^2 ^(CEU) and 997,694 SNP pairs for which *r*^2 ^(AJ) <*r*^2 ^(CEU), and the sign test indicated that there was a significant excess of LD in AJ relative to CEU (*P *< 10^-10^). Restricting attention to only *r*^2 ^values calculated between each SNP and its nearest neighbor (higher order LD imposes a non-independence across LD values), a different pattern was observed. Here there was significantly higher LD in CEU than AJ, where *r*^2 ^(AJ) > *r*^2 ^(CEU) for 11,228 tests and *r*^2 ^(AJ) <*r*^2 ^(CEU) for 13,391 tests (*P *< 10^-10^). This pattern was consistent with our analysis of LD decay, where CEU had higher levels of LD for SNPs close to each other. However, there did appear to be a trend toward higher *r*^2 ^for AJ compared to CEU when considering only SNP pairs separated by greater than 200 kb, where *r*^2 ^(AJ) <*r*^2 ^(CEU) for 13,609 tests and *r*^2 ^(AJ) > *r*^2 ^(CEU) for 14,570 tests (*P *< 10^-8^). These results can be explained in terms of the sampling variance of LD caused by a founder event (see Discussion).

**Figure 6:**
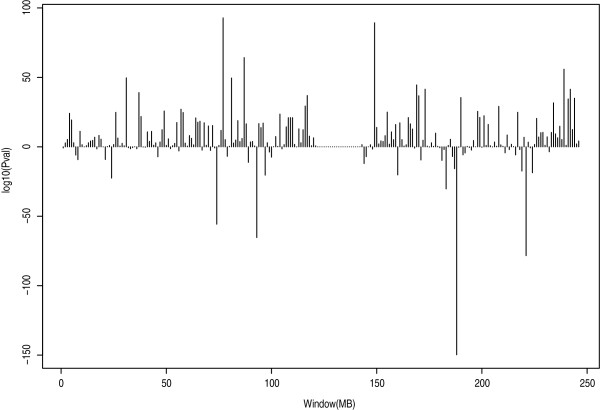
*P*-values from a sign test plotted for each 1 Mb region of chromosome 1. Data were drawn from a random sample replicate of 60 AJ compared to 60 CEU. A positive value indicates greater LD in AJ compared to CEU.

The same procedure was used for all SNPs within each 1 Mb window slid along chromosome 1 in order to identify local regions with LD differences between the populations. The sign test plotted for each 1 Mb region of chromosome 1 for one representative sample of 60 AJ resulted in the *P*-values shown in Fig. 6. Patterns of LD were quite variable across the chromosome, with AJ having higher LD at 73–74 Mb and CEU having higher LD at 187–188 Mb. Also examined was the number of 1 Mb bins on chromosome 1 where the sign test was significant (*P *< 0.05) for an excess of LD in one population or the other. This analysis was performed separately for each of the six subsamples of 60 AJ individuals. When considering all SNPs, CEU had higher LD than AJ for the region 42–49 Mb, and AJ had higher LD than CEU for 125–133 Mb. When considering pairs of SNPs and their closest neighbor, CEU had higher LD than AJ for 38–52 Mb and AJ had greater LD for 0–2 Mb. Finally, when considering pairs of SNPs at least 200 kb apart, CEU had higher LD than AJ for 14–18 Mb while AJ had higher LD for 26–37 Mb windows. These results appear to reflect the stochastic nature of sampling that occurred at founding of the ancestral AJ population. To assess the impact of selecting different samples of 60 AJ, the number of times that one population had significantly higher LD with one sample and the opposite population had significantly higher LD with a different sample was examined. For all SNPs, the average number of 1 Mb windows where this switch occurred was 8.2. For neighboring SNPs, no windows switched and for SNPs 200 kb apart, an average of 0.267 windows switched.

To compare these data to those from a recent analysis of LD structure on chromosome 22 [36], a sliding window plot of average *r*^2 ^and 1.7 Mb windows with a 1.6 Mb overlap was utilized, in contrast to the 1 Mb non-overlapping windows used in this study. The pattern of average *r*^2 ^peaks was similar to that noted in the previous study (Additional File 7, Figure S6). The sign tests for chromosome 22 were qualitatively similar to those on chromosome 1. On chromosome 22, there were 194,639 SNP pairs for which *r*^2 ^(AJ) > *r*^2 ^(CEU) and 179,166 SNP pairs for which *r*^2 ^(AJ) <*r*^2 ^(CEU), and the sign test indicated that there was a significant excess of LD in AJ relative to CEU (*P *< 10^-10^). For independent pairs of neighboring SNPs there was significantly higher LD in CEU than AJ, where *r*^2 ^(AJ) > *r*^2 ^(CEU) for 1,951 tests and *r*^2 ^(AJ) <*r*^2 ^(CEU) for 2,188 tests (*P *< 0.00025). When considering only independent SNP pairs separated by greater than 200 kb, there was a significant excess of LD in AJ relative to CEU. There were 2,145 tests where *r*^2 ^(AJ) <*r*^2 ^(CEU) and 2,392 tests where *r*^2 ^(AJ) > *r*^2 ^(CEU) (*P *< 0.00026). Chromosome 22 also showed local regions where LD was significantly different between the AJ and CEU samples: when considering all pairs of SNPs there were 21 to 26 1-Mb windows where AJ had significantly higher LD than CEU, and three to six 1-Mb windows where CEU had significantly higher LD than AJ.

As an additional metric to compare LD between the two populations, we looked at the proportion of SNP pairs with no evidence of recombination (*D' *= 1.0) at different distance intervals between the SNPs, as previously described Shifman et al. 2003 [37]. In this analysis, CEU had more LD at closer (< 200 kb) distances between SNPs, while AJ had slightly higher LD at longer distances between SNPs (Additional File 8, Figure S7).

**Figure 7:**
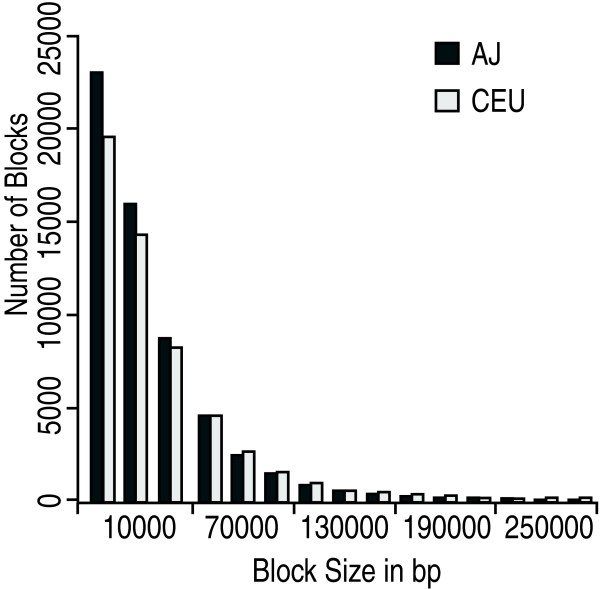
Histograms of autosome block sizes estimated using HAP for AJ and CEU. X chromosome block calculations were excluded from the histogram in order to minimize reliance upon haplotype estimates from the thirty CEU founder males. A small number of outlier blocks greater than 300 kb were also excluded from the figure.

The HAP-derived haplotype block estimates gave 59,269 blocks for AJ and 54,457 blocks for CEU. Since this method puts every SNP in a block, more blocks indicates that each block is smaller. This is reflected in AJ blocks having an average length of 36.8 kb (median 26.6 kb), while CEU blocks had an average length of 40.6 kb (median 28.9 kb). The distribution of block lengths is displayed in Fig. 7. Similarly, the number of tagged SNPs was 136,584 in AJ (average of 2.3 per block), and 128,205 in CEU (average of 2.4 per block). HAP derived haplotype blocks by chromosome are voluminous data available upon request from the authors. Since there were 101 AJ individuals and 60 CEU individuals, we examined haplotypes using only 60 AJ, but the results were similar to those obtained using all the samples (data not shown). Once the single SNP blocks were removed, the average and median blocks were still smaller in AJ. The average length was 38.0 kb in AJ compare to 42.1 kb in CEU and the median was 27.7 kb in AJ compared to 30.3 kb in CEU.

Haplotype blocks were also estimated using the method of Gabriel et al. [28] implemented in Haploview. There were 50,397 AJ blocks and 44,169 CEU blocks. This agrees with the HAP analysis in that there are more blocks in AJ compared to CEU. The average block length for AJ was 26.5 kb (median 11.3 kb), which was less than the average of 27.5 kb (median 12.3 kb) for CEU. The shorter block lengths in AJ were consistent with the HAP results.

### Homozygosity mapping

**Figure 8:**
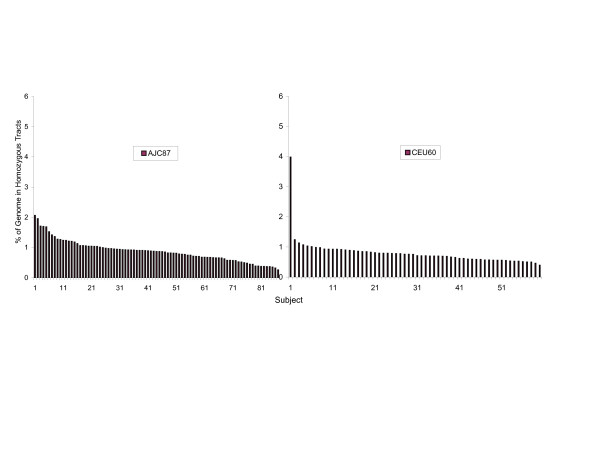
Frequency plot of the percentage of the genome within homozygous tracts. The percentage is shown for each of 101 AJ (**a**) and 60 CEU (**b**).

The median number of homozygous tracts per individual reaching the threshold score of 20 was 26 for AJ and 28 for CEU. The median tract lengths were significantly different, at 731 kb in AJ and 621 kb in CEU (*P *< 10^-10^). A significantly greater proportion of the AJ genome was contained in homozygous tracts (median 0.87%; range 0.26% – 2.07%) compared to CEU (median 0.73%; range 0.39% – 3.98%) (*P *= 0.02) suggesting a greater level of autozygosity in AJ (Fig. 8). There was one clear outlier in the CEU population, sample NA12874, which was also identified in a previous study of homozygosity tracts as an outlier [38].

### Principal components analysis

In order to compare the two populations by reducing dimensionality to two axes according to the Principal Components Analysis (pca) implementation of Price et al., [32] we first performed pca analysis on all 435,632 SNPs in the CEU and AJ overlapping sets as well as subset of 167,676 SNPs with no detectable Hardy Weinberg departures and no call rates below 1%. Both analyses revealed discrete clustering of the populations, as shown in Fig, 9, consistent with the F_*ST *_findings. Some outliers were evident among the AJ set, consistent with outbreeding. Subsequent analyses were performed on the entire SNP set in order to include a full representation as in recent association studies [39], but re-analysis was also performed on the subset of 87 cases left after removal of 14 subjects identified by pca analysis. For this subset there were 28,053 SNPs with call rates below 95% and 9,395 with call rates below 90% in the unfiltered analysis of 435,632 SNPs, but in the filtered analysis of 167,676 SNPs all rates were >99.6%. For the LD decay analysis, this recalculation showed that for SNPs <150–200 kb apart, the CEU have more LD, while for SNPs >200 kb apart, the AJ had slightly more LD. This was seen using both % of SNP pairs where *D*' = 1 as well as average *r*^2^. The average *r*^2 ^values differ slightly from using subsets from the 101 AJ, but the overall patterns remained the same. When the 14 PCA outliers were removed, we found that the mean F_*ST*_, representing the distance between the AJ and CEU increased slightly, to 0.0099 from 0.0094. In addition, the mean heterozygosity increased slightly when outlier AJ were removed, from 0.26 to 0.28. However, the mean heterozygosity was not significantly different in either case from the CEU, which was 0.27.

**Figure 9:**
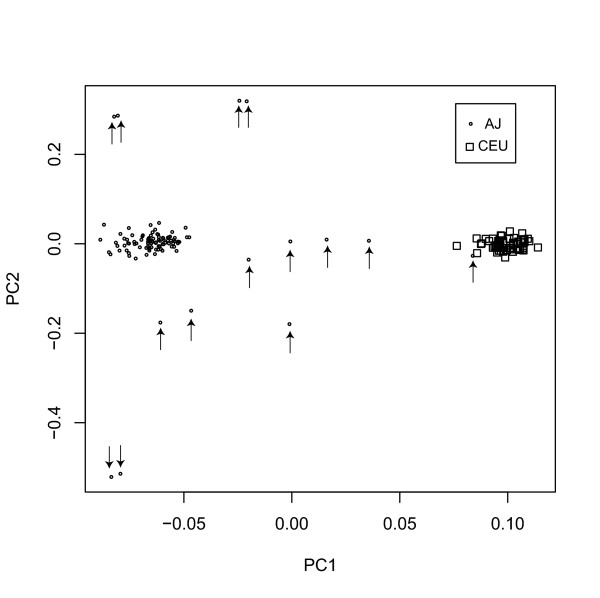
Scatter plot of principal components analysis of 435,632 SNPs. Principal component 1 is plotted on the abscissa, component 2 on the ordinate; CEU are designated as open boxes and AJ as smaller open circles. One AJ can be observed in the CEU cluster. Those case removed from analysis following PCA are denoted by an arrow.

## Discussion

A high density genomic scan was used to compare several aspects of the structure of genetic variation in a genetic isolate (AJ), and a representative population of northern European ancestry (CEU). Prior studies of haploid regions of the genome, including mitochondrial DNA and the nonrecombining portion of the Y chromosome, support the hypothesis that AJ are genetically distinct, with little recent admixture with host European populations [40-43]. While the relative endogamy of AJ has been postulated based on historical documentation of population bottlenecks and the isolation of AJ in Europe, initial analysis of LD structure in AJ compared to Europeans showed modest increases in LD [36,37]. In those studies, comparisons between AJ and Europeans were made based on LD in two 1 Mb regions per chromosome, typed with 16 SNPs per region [37], or were restricted to a single chromosome [36]. In contrast to these studies which utilized about one SNP per 62.5 kb across the genome [37] or 2,589 SNPs at a density of one marker per 13.8 kb on a single chromosome [36], in the current study, a high density analysis of genetic variation in AJ was performed utilizing 435,632 SNPs at a mean density of one SNP per 2.5 kb (median one SNP per 5.8 kb) across the entire genome. While our study also found regions where AJ show greater LD than CEU (e.g. the region analyzed by Service et al. [36]), LD structure was highly variable with CEU showing greater measure of LD on other chromosomes.

Our analysis reveals small but significant differences in measures of genetic diversity between AJ and CEU. The mean value of *F*_*ST*_, a useful measure of overall genetic divergence among subpopulations, was only 0.009, but because of the large number of SNPs typed, this small value is nevertheless highly unlikely to occur by chance (*P *< 0.001). The biological interpretation of *F*_*ST *_values as high as 0.05 generally indicate negligible genetic differentiation [44,45], underscoring the power of dense SNP genotyping for detecting evidence of historical isolation despite small genetic differences. The areas of greatest difference as assessed by *F*_*ST *_were on chromosome 2 and chromosome 6, presumably involving the HLA region on chromosome 6 and the LCT locus on chromosome 2. The differences in the HLA regions are also consistent with the SNP-by-SNP comparisons. Distinct patterns of LD in the HLA region have been observed in AJ [46] and selective sweeps at LCT have been shown in European populations [47]. Associations have been made between specific HLA alleles and several disorders [48-50]. Very recently, a consortium group has derived an *F*_*ST *_value comparing Northern Europeans to Ashkenazi Jews, also on the Affymetrix 500 K platform [51]. They report a value of 0.009, identical to that observed here. Together this study and the current study begin to create an Ashkenazi specific "HapMap" and also begin to define subsets of markers sufficient to distinguish these populations.

Because of the founder effect, inflated sampling variance of haplotype frequencies in turn results in inflated variance in LD. For SNPs that are very close to one another, if the parent population had high LD, the AJ could witness lower LD due to this sampling. Most SNPs at somewhat greater distances would have had nearly zero LD, and it is the founding effect that produced the observed greater LD observed in AJ at intermediate SNP distances. Because haplotype blocks are generally inferred from the relatively close, high-mutual LD set, this sampling variance could be expected to erode the size of these blocks. When actual measures of genome-wide LD structure in the two populations were compared, haplotype blocks inferred from pair-wise LD statistics as well as by EM haplotype phase inference did indeed tend to be smaller in AJ. Analysis of global LD decay showed essentially no difference between AJ and CEU, although there was a tendency for faster decay of nearby SNPs and slower decay of intermediate distance SNPs in the AJ. These data are more consistent with the AJ as an older, larger population than CEU, and would suggest that the LD structure of AJ may not provide a global advantage for whole-genome association mapping. In contrast, however, the proportion of SNP pairs in CEU showing no evidence of recombination (*D' *= 1) among SNP pairs at different distance intervals was greater in CEU than in AJ only at short distances, with the AJ generally showing more LD at longer distances. Similarly, analysis of local LD, for example the analysis of LD decay at chromosome 1, revealed a similar pattern, with AJ showing slower decay (more LD) at longer distances. In addition, a likelihood ratio approach showed that runs of homozygous SNPs were approximately 25% longer in AJ than CEU, which is more consistent with expectation in a genetic isolate. These aggregate data suggest that CEU, like AJ, underwent a population bottleneck, but given that the overall diversity of AJ is not lower than that of CEU, the greater homozygous tract lengths in AJ imply an average shorter time back to common ancestry for the AJ sample.

The data presented here demonstrate that founder effect advantages for AJ as applied to LD mapping will be regionally variable. A recent analysis of LD structure utilizing 2,486 SNPs on chromosome 22 [36] revealed generally greater average *r*^2 ^in AJ individuals, leading to the conclusion that association analyses in groups like AJ would require at least 30% fewer markers than studies in outbred populations [36]. The analysis reported herein, based on more than twice the SNP density, revealed a pattern of LD on chromosome 22 that is virtually identical to that observed by Service et al[36]. However, analysis of other loci (e.g., chromosome 1) as well as a global genome analysis revealed significant variability in local LD structure. Thus, in undertaking LD mapping for gene discovery in AJ, regional variability lending a founder effect advantage will occur in only some regions of the genome.

To explore the basis of the differences in LD structure noted here, it is important to consider possible sources of ascertainment bias resulting from the selection of AJ subjects or those in the comparison CEU group. In this study we utilized an American AJ cohort of women without a history of breast cancer, while a prior study utilized an Israeli AJ cohort [37]. Shifman et al. [37] found very high similarity of allele frequencies (*r*^2 ^= 0.96) comparing AJ and Caucasian individuals. In contrast, an *r*^2 ^of 0.83 was observed in this study. This latter value did not change using major allele frequencies, or if SNPs were filtered based on HWE violations by Fisher's exact test or Spearman's Rho test. These findings suggest possible biases due to population stratification, or alternatively, that the genomic measures employed more accurately reflect true allele frequency differences than in the prior study. Notably, the American AJ samples used here and the Israeli AJ samples in the ascertainment of Shifman et al. [37] appear to have similar proportions of SNP pairs showing no evidence of recombination (*D*' = 1) for pairs less than 5 kb apart (81% in our U.S. samples versus 76% in the prior series). This suggests a general comparability in the AJ sample sets with regard to LD structure.

However, for the comparison group of "European" samples, there were striking differences; the proportions of SNP pairs showing no evidence of recombination (*D*' = 1.0) for pairs less than 5 kb apart was 86% in our series versus 63% in the prior series. All or part of the comparison ascertainment in the prior series' [36,37] samples was from the NIGMS Human Genetic Cell Repository at Coriell, whereas we utilized the CEPH reference families derived from Utah residents of European ancestry. It is therefore possible that differences in our findings and those of prior studies are a result of these differing ascertainments of those of European ancestry. Because of polygyny and founder effects associated with the Utah Mormon genealogies [52,53], this population may not serve as a representative European comparison group. However, gene frequency data, including red cell antigen and HLA loci, were similar between CEU and northern European cohorts in an early study [52]. Similarly, a more recent study of LD structure showed nearly identical patterns, although only regions comprising 14.3 Mb of the genome were compared [54].

The data presented here are consistent with a hypothesis that the high level of similarity of patterns of LD between AJ and CEU results from the same historical events that shaped the extended LD in these two populations. The finding of regions of slower LD decay (greater LD) in AJ for distant SNPs is not readily explained, and suggests the possibility of ancestral admixture. It is clear that regions of LD around pathogenic mutations in AJ can be quite large, extending up to 10 Mb, consistent with their more recent origin. While the historical record is subject to interpretation, demographic considerations and analysis of coalescence times of founder mutations suggest at least three periods of founding and expansion of AJ, one greater than 100 generations (20 centuries) ago, marking the founding and expansion of the Jewish population in the Middle East, one approximately 20 generations (five centuries) ago [8,9], marking a constriction resulting from persecution and the Plague, and subsequent expansion of AJ in central Europe, and, finally, a more recent event approximately 12 generations (three centuries) ago marking the constriction of AJ in Europe as a result of renewed persecution, and subsequent re-expansion.

Not all founder mutations in AJ resulted from the most recent bottlenecks; the I1307 K allele of *APC*, for example, seen in both Sephardic as well as Ashkenazi Jews, dates to the initial bottleneck from 100 generations ago [9,55]. Given these demographic and historical observations, it is perhaps not surprising that the LD map of the Ashkenazim reveals features both of its ancient origins (a greater number of smaller sized haplotype blocks) but also greater endogamy (increased size of homozygous regions identical by decent) compared to Europeans. It is also likely that the local genomic differences observed between AJ and CEU (regional differences in *F*_*st *_and local differences in LD decay) reflect the impact of both selection as well as genetic drift. Analysis of specific regions of local difference by tests of evolutionary neutrality will be needed to explore selective effects, which have recently been documented in European, Chinese, and African populations [56]. The predominant impact of founder effects in AJ, however, is evidenced by the documentation of pathogenic mutations for more than 20 heritable diseases, including heterozygous syndromes (e.g. hereditary breast and ovarian cancer), where there is less precedent for selective advantage than for carriers of recessive traits [8,10].

Whether the genetic characteristics of AJ, revealed here to be complex and showing local differences in LD structure, will prove to be helpful in genomic association studies remains to be determined. Based on computer modeling, it has been demonstrated that if haplotypes were introduced by a small number of founders, LD will be greater in isolated compared to outbred populations [57]. In the case of extreme genetic isolates, extended LD was clearly evident around even common alleles when compared with neighboring populations [58]. While clearly advantageous for mapping rare alleles with population frequencies less than the reciprocal of the effective number of founding chromosomes, initial experience indicates that gene mapping advantages may be limited in discovering alleles associated with complex disease [4]. In that setting, rare mutations may be present on an extended haplotype, as a result of one or several original founding chromosomes carrying the particular mutation. More common alleles may also enter small founder populations multiple times resulting in lengths of shared haplotypes around these alleles that are indistinguishable from the larger ancestral population [57].

Despite these potential limitations of LD mapping in AJ, SNP-based LD mapping successfully "rediscovered" *BLM *and *BRCA2 *in proof-of-principle exercises using AJ cohorts [11,12]. While genomic association studies in large outbred populations are seeking to map loci for common cancer susceptibility genes, it remains to be seen if this same approach using AJ will benefit from local increases in LD around candidate loci. Based on this preliminary LD map of AJ, the advantage of genome-wide association studies in AJ compared to CEU are likely to be modest and highly dependent on regional LD structure.

## Conclusion

There were small but significant differences in measures of genetic diversity between AJ and CEU. Analysis of genome-wide LD structure revealed a greater number of haplotype blocks which tended to be smaller in AJ. There was essentially no difference in global LD decay between AJ and CEU, although there was a tendency for faster decay of nearby SNPs and slower decay of intermediate distance SNPs in the AJ. These data are more consistent with the AJ as an older, larger population than CEU, and would suggest that, depending on regional differences in LD structure, AJ populations may not always provide an advantage for whole-genome association mapping.

## Authors' contributions

ABO participated in the design of the study, data analysis, and drafted the manuscript.

BG participated in the design of the study, data contribution/assembly, data analysis, and drafted the manuscript.

KEL contributed to data analysis.

JPS participated in data contribution/assembly and data analysis.

JS contributed to data analysis and drafted the manuscript.

SAS contributed to data analysis.

EE contributed to data analysis.

TK participated in data contribution/assembly and data analysis.

JAL contributed to data analysis.

RJK contributed to data analysis.

EF participated in data contribution/assembly.

LN participated in the design of the study and contributed to the financial support.

NAE participated in the design of the study and data analysis.

AV participated in data contribution/assembly and data analysis.

CSL contributed to data analysis.

PIB contributed to the financial support.

AGC contributed to data analysis and drafted the manuscript.

KO participated in the design of the study, drafted the manuscript, and contributed to the financial support.

JB participated in the design of the study and contributed to the financial support and drafting of the manuscript.

All authors read and approved the final manuscript.

## Supplementary Material

Additional File 1 Table S1. Distribution of SNPs on each chromosome arm Click here for file

Additional File 2 Figure S1.Percentage of No Calls per SNP by array type Click here for file

Additional File 3 Figure S2. Percentage of No Calls per SNP by array type for SNPS with completion rate of greater than 99.6% Click here for file

Additional File 4 Figure S3. Percentage of No Calls per SNP by array type in CEU Click here for file

Additional File 5 Figure S4. Percentage of No Calls per SNP by array type in CEU Click here for file

Additional File 6 Figure S5. Analysis of Hardy-Weinberg Departures Click here for file

Additional File 7 Figure S6. A sliding window plot of average *r*^2 ^values across chromosome 22. For this analysis, 1.7 Mb windows with a 1.6 Mb overlap were utilized. Click here for file

Additional File 8 Figure S7. The proportion of SNPs with *D*' = 1 as a function of distance between SNPs, with data drawn from chromosome 1. Click here for file
